# Lithium-coupled electron transfer reactions of nano-confined WO_x_ within Zr-based metal–organic framework

**DOI:** 10.3389/fchem.2024.1427536

**Published:** 2024-06-14

**Authors:** Hafsa Abdul Ghuffar, Hyunho Noh

**Affiliations:** Department of Chemistry and Biochemistry, The University of Oklahoma, Norman, OK, United States

**Keywords:** metal-organic framework, Li-coupled electron transfer, Li-ion battery, electrochemistry, thermochemistry

## Abstract

Interfacial charge transfer reactions involving cations and electrons are fundamental to (photo/electro) catalysis, energy storage, and beyond. Lithium-coupled electron transfer (LCET) at the electrode-electrolyte interfaces of lithium-ion batteries (LIBs) is a preeminent example to highlight the importance of charge transfer in modern-day society. The thermodynamics of LCET reactions define the minimal energy for charge/discharge of LIBs, and yet, these parameters are rarely available in the literature. Here, we demonstrate the successful incorporation of tungsten oxides (WO_x_) within a chemically stable Zr-based metal−organic framework (MOF), MOF-808. Cyclic voltammograms (CVs) of the composite, WO_x_@MOF-808, in Li^+^-containing acetonitrile (MeCN)-based electrolytes showed an irreversible, cathodic Faradaic feature that shifted in a Nernstian fashion with respect to the Li^+^ concentration, i.e., ∼59 mV/log [(Li^+^)]. The Nernstian dependence established 1:1 stoichiometry of Li^+^ and e^−^. Using the standard redox potential of Li^+/0^, the apparent free energy of lithiation of WO_x_@MOF-808 (ΔG_app,Li_) was calculated to be −36 ± 1 kcal mol^−1^. ΔG_app,Li_ is an *intrinsic* parameter of WO_x_@MOF-808, and thus by deriving the similar reaction free energies of other metal oxides, their direct comparisons can be achieved. Implications of the reported measurements will be further contrasted to proton-coupled electron transfer (PCET) reactions on metal oxides.

## 1 Introduction

Cation-coupled electron transfer reactions at electrode-electrolyte interfaces are critical to modern-day electrolyzers, (photo) electrocatalysts, batteries, and many others. These serve as the core technologies to shift the energy and chemical sectors away from fossil fuels to those that are more renewable, such as solar and wind energy (Adams et al., 2003; Peper and Mayer, 2019; Prather et al., 2023). Nearly all charge transfer reactions relevant to renewable energy involve interfacial electron transfer reactions that are coupled with cations. Perhaps the most seminal example of coupled charge transfer reaction occurs within lithium-ion batteries (LIBs). Electrons transferred between the anode (graphite) and the cathode (layered metal oxides) are coupled with lithium cations in the electrolyte. Eq. 1 below shows the lithium-coupled electron transfer (LCET) reaction at the metal oxide (M^n+^O_x_) cathode (Van Noorden, 2014; Kim et al., 2019; Manthiram, 2020).

$$M^{\left(n+\right)}O_{x\;\left(s\right)}=y{\text{Li}}_{\;\left(\text{solv}\right)}^{+}+{\text{ze}}^{-}⇌{\text{Li}}_{y}M^{\left(n-z\right)+}O_{x\;\left(s\right)}$$ (1)

**FIGURE 1:**
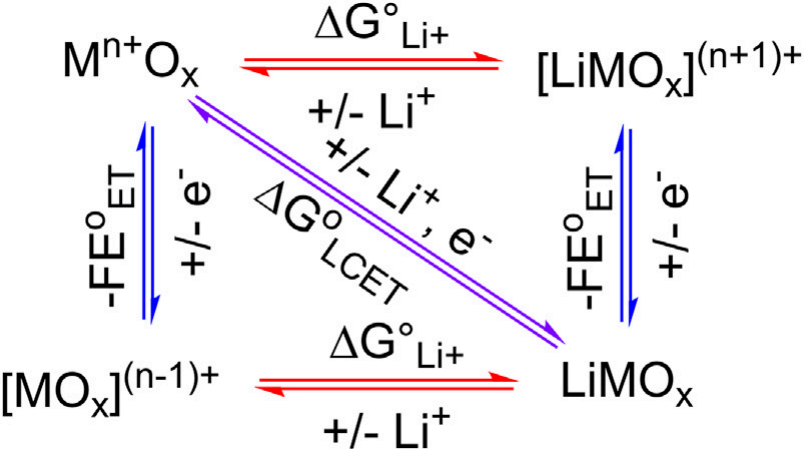
Square scheme of LCET. Here, 1:1 Li-to-electron stoichiometry is assumed.

In surface science, electrocatalysis, and energy storage literature, it is often *implicitly* assumed that the stoichiometry of the involved cation and the electron is equal, i.e., in Eq. 1, y = z (Poizot et al., 2002; Choi et al., 2003; Kim et al., 2019; Mayur et al., 2019; Manthiram, 2020). This is self-evident from the square scheme of an LCET reaction using metal oxide (Figure 1), as equimolar amounts of cation and electron prevent the formation of charged, and therefore energetically unfavorable species. Computational modeling of TiO_2_ clusters further elucidated an increase in the equilibrium constant (*K*) of LCET reaction by 10^8^ upon the addition of a single electron (Zhang et al., 2012). However, for many metal oxides that undergo proton-coupled electron transfer (PCET) reactions, redox reactions can involve more than one proton per electron (Burke and Lyons, 1986; Dincă et al., 2010; Gambardella et al., 2011; Fleischmann et al., 2020; Mayer, 2023). Thus, we argue that the equal stoichiometry of LCET is also not guaranteed and cannot be assumed.

The Li^+^-to-electron stoichiometry is fundamental in deriving the thermodynamics of the LCET reaction, which defines the *minimal energy* required to charge and discharge electrical energy in LIBs. This is conceptually analogous to the thermodynamic potential of an electrocatalytic reaction (Bard and Faulkner, 2001). Optimal electrocatalysts exhibit high catalytic activity at the thermodynamic potential (*E* ° ), minimizing the energetic cost (Medford et al., 2015; Seh et al., 2017). In electrocatalysis that involves PCET reactions like the reactions of H_2_, O_2_, CO_2_, and many others, the formal potential (*E* ° ’) values shift as a function of the proton activity of the reaction medium; when equimolar amounts of protons and electrons are involved in the overall reaction, this shift should be close to 59 mV per unit change in pH (Mayer, 2004; Agarwal et al., 2021; Nocera, 2022). In the same way, *E* ° ’ of LCET reaction should shift as a function of the Li^+^ concentration; Eq. 2 is the Nernst equation for the LCET reaction of a generic metal oxide, M^n+^O_x_ shown in Eq. 1. Because this is a redox reaction of heterogenized species, fractional surface coverages (*θ*) of reduced and oxidized metal oxides are used instead of the concentrations (Ingram et al., 2024).

$$E=E{}^{\circ}-\frac{0.059}{z}\log\left(\frac{\theta_{{Li}_{y}M^{\left(n-z\right)+}O_{x}}}{\theta_{MO_{x}}}\right)-0.059\left(\frac{y}{z}\right)\log\left(\left[{Li}^{+}\right]\right)$$ (2)

To this day, reports on LCET reaction thermodynamics remain rare in the literature, particularly when compared to those related to PCET reactions. Free energies of PCET reactions have long been examined both experimentally and computationally on molecular species, metals, and even binary and ternary material surfaces (Nørskov et al., 2005; Strmcnik et al., 2008; Seh et al., 2017; Wise et al., 2020; Agarwal et al., 2021; Fertig et al., 2021; Noh and Mayer, 2022; Fortunato et al., 2023; Nedzbala et al., 2024). A few reports related to LCET of cobalt, nickel, vanadium, tungsten, and many other oxides suggest that the reaction free energy (ΔG ° _LCET_) is dependent on synthesis, structure, morphologies, chemical history, and many other parameters (Okubo et al., 2007; Kerisit et al., 2009; Pan et al., 2010; Wang et al., 2016; Ng et al., 2020). However, the ΔG ° _LCET_ and their dependence on the physical/chemical environment remain to be determined.

**FIGURE 2:**
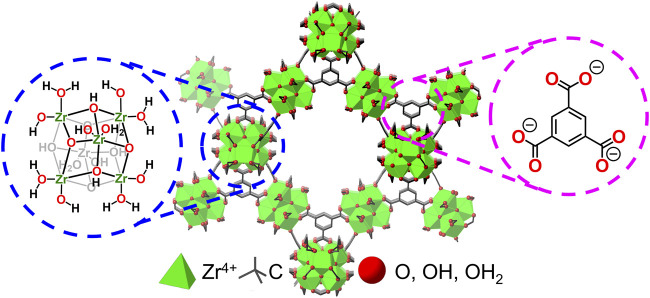
Crystal structure of Zr-based MOF, MOF-808, and its inorganic node and the organic linker.

Herein, we report the successful incorporation of tungsten oxides (WO_x_) that undergo LCET reactions within the pores of the Zr-based metal−organic framework, MOF-808 (the MOF-WO_x_ composite here onwards will be referred to as WO_x_@MOF-808; see Figure 2 for the parent MOF structure) (Furukawa et al., 2014). MOF-808 and many other MOFs have been widely applied in the field of energy and environmental applications (Zhang et al., 2017; Zhao et al., 2020; Shi et al., 2024). Hydrated tungsten oxides are of particular interest as they have been demonstrated to undergo Faradaic reactions in the presence of protons, lithium, and other cations (He et al., 2016; Mitchell et al., 2017; Zheng et al., 2018; Mitchell et al., 2019). The chemical stability of Zr-based MOFs ensures that the porous MOF backbone remains intact throughout all measurements (Bai et al., 2016; Howarth et al., 2016; Shi et al., 2023). The electrochemical and thermochemical analysis of the MOF pore-confined WO_x_ should be pivotal to understanding the fundamental role of the physical properties of electrodes on LCET thermodynamics, alluding to the design principles of energy storage materials.

## 2 Materials and methods

### 2.1 Synthesis of WO_x_@MOF-808

Zr-based MOF-808 was synthesized and thermally activated according to the reported procedure (Liu et al., 2021). Subsequent incorporation of WO_x_ within the MOF pores through acid precipitation described below is modified from that reported by Freedman (Freedman, 1959). Briefly, freshly synthesized Zr-MOF-808 (12 mg) was submerged into 1 mL of a pH 7-adjusted aqueous buffer consisting of 100 mM of 3-(*N*-morpholino)propanesulfonic acid (MOPS). Under vigorous stirring, 16.4 mg of Na_2_WO_4_ ∙ 2H_2_O was added to the reaction mixture; this is equivalent to ∼5.4 eq. of [WO_4_]^2−^ with respect to the Zr_6_ node within MOF-808. The reaction was left stirred for 1 h and was centrifuged to isolate the MOF composite and was further exposed to 1 M HCl overnight. The color change of the MOF composite from white to yellow indicated the successful synthesis of WO_x_@MOF-808. The sample was further washed with H_2_O and acetone and was activated at 80 ° C under dynamic vacuum. The sample porosity, morphology, crystallinity, and the WO_x_ loading were confirmed through N_2_ adsorption-desorption isotherm, scanning electron microscopy coupled with energy dispersive X-ray spectroscopy (SEM-EDS), and powder X-ray diffraction (PXRD) patterns; see the Supplementary Material for details.

### 2.2 Electrochemical measurements in acetonitrile

All electrochemical measurements of WO_x_@MOF-808 were measured in a MeCN-based electrolyte that contains LiClO_4_ with concentrations ranging between 25 and 250 mM. The total ionic strength of the electrolyte was kept consistent at 1 M using TBAClO_4_. Pt wire and Ag/Ag^+^ were used as the counter and pseudoreference electrodes, respectively. A small amount of ferrocene (Fc) was added at the end of each experiment to calibrate the measured electrochemical potential. Unless otherwise noted, all cyclic voltammograms (CVs) were measured at a scan rate (υ) of 100 mV/s.

## 3 Results

### 3.1 Physical characterization of WO_x_@MOF-808

**FIGURE 3:**
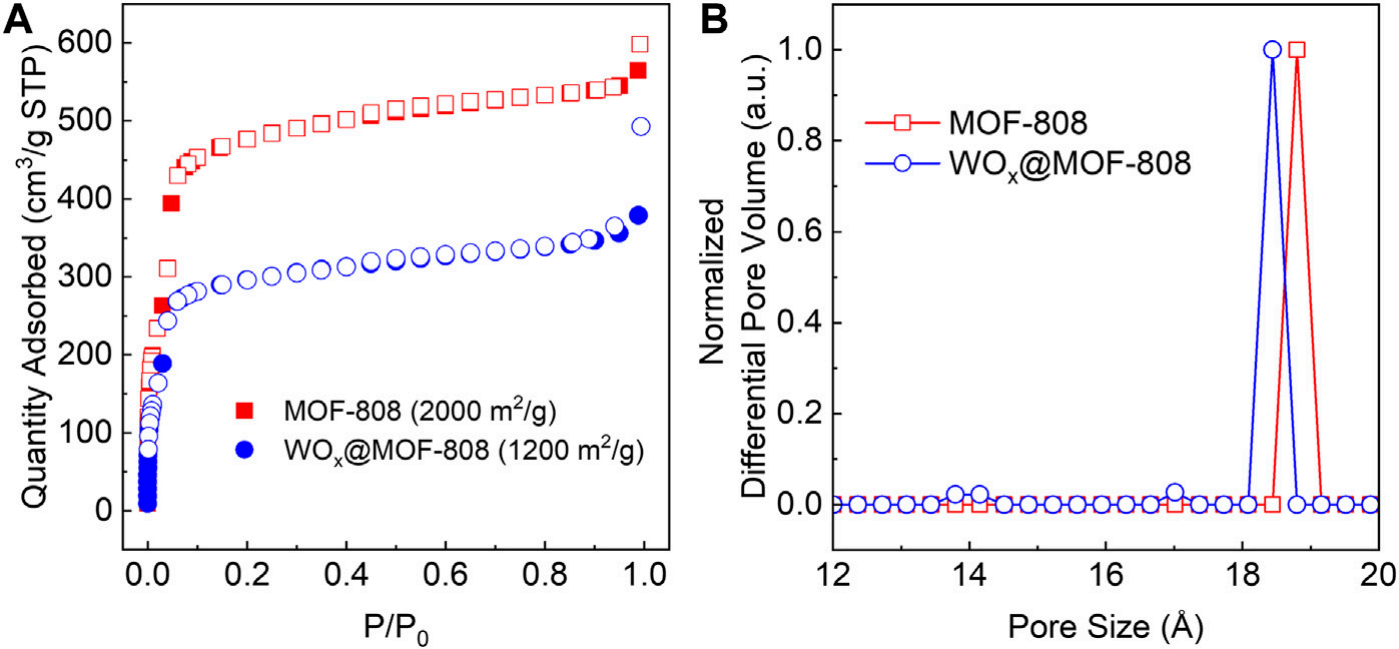
**(A)** N_2_-adsorption desorption isotherm and **(B)** DFT-calculated pore size distributions of pristine MOF-808 and WOx@MOF-808.

N_2_-adsorption-desorption isotherm of WO_x_@MOF-808 exhibited a significant decrease in the N_2_ uptake, resulting in a decrease in the Brauner-Emmett-Teller (BET) area from 2,000 to 1,200 m^2^/g (Figure 3A). This decrease can be attributed to 1) the incorporation of WO_x_ within otherwise vacant MOF pores or 2) an increase in molar mass. Pristine MOF-808 has a pore size of 18.8 Å; this pore decreased to 18.4 Å upon WO_x_ incorporation. Post-synthetic WO_x_ deposition also led to the formation of pores with diameters of ∼14 and 17 Å (Figure 3B). Scanning electron microscopy (SEM) images of MOF-808 and WO_x_@MOF-808 (Supplementary Figure S2) show that after WO_x_ incorporation, the surfaces of the MOF crystallites are roughened, which may be due to surface WO_x_ or due to the acidic tungstic acid present during the synthesis of WO_x_@MOF-808 (see Section 4.1 for more details). Energy dispersive X-ray spectroscopy (EDS) revealed that essentially all [WO_4_]^2−^ were incorporated into the MOF network per Zr_6_ node, nearly doubling the molecular mass; the molar masses of MOF-808 and WO_x_@MOF-808 are 1,303 and at least 2,400 g/mol, respectively (Furukawa et al., 2014). The apparent decrease in N_2_ uptake can be ascribed to both factors. The powder X-ray diffraction pattern (PXRD) confirmed that the bulk crystallinity of the MOF backbone was retained during the WO_x_ incorporation (Supplementary Figure S3).

### 3.2 Cyclic voltammograms (CVs) of WO_x_@MOF-808 in Li-containing MeCN

**FIGURE 4:**
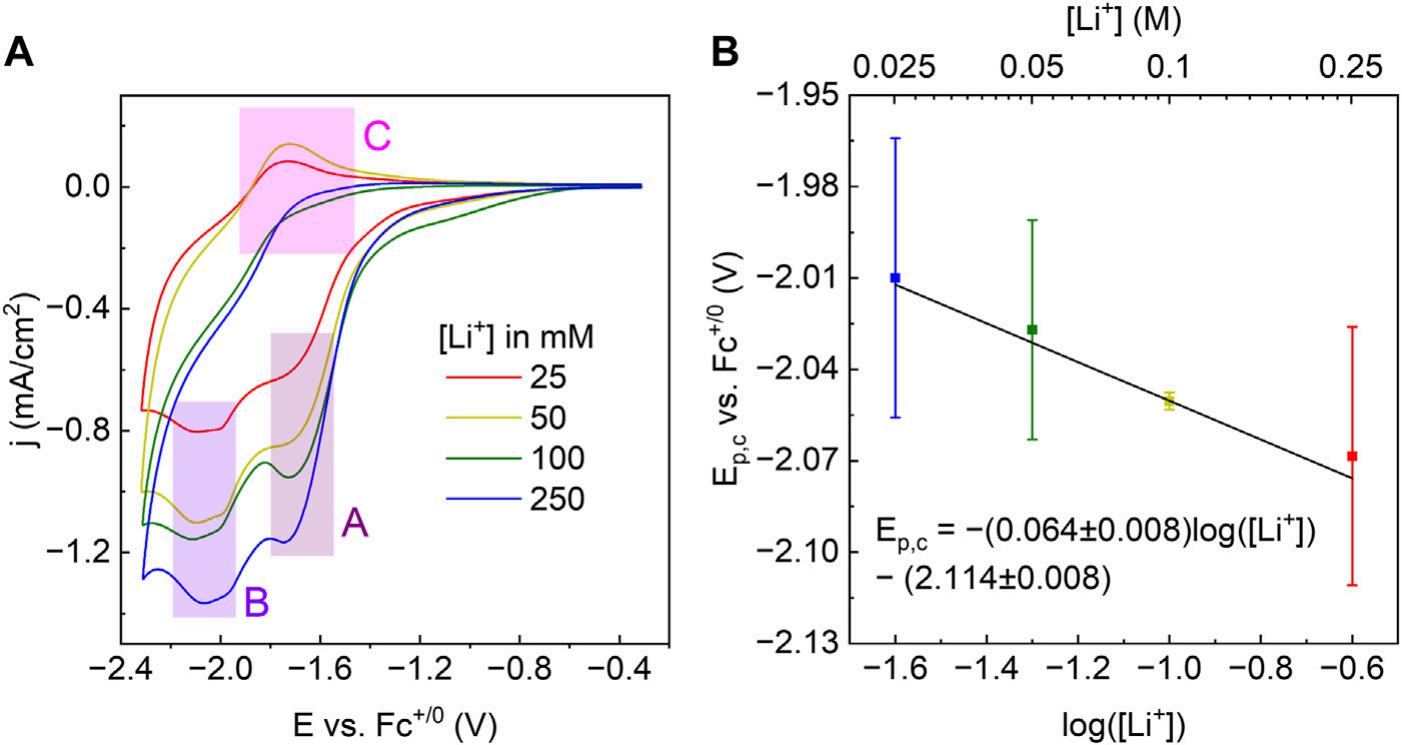
**(A)** CVs of WO_x_@MOF-808 in various concentrations of Li-ions. **(B)** Plot of E_p,c_ of feature labeled B in **(A)** against the log of [Li^+^].

CVs of WO_x_@MOF-808 in LiClO_4_/TBAClO_4_-containing electrolyte showed two cathodic and one anodic features (Figure 4A). Amongst the three Faradaic features, the second cathodic feature (labeled B in Figure 4A) was ascribed to the LCET reaction of pore-confined WO_x_. The peak cathodic potential (E_p,c_) scaled in a roughly Nernstian fashion with respect to the concentration of Li^+^ in the electrolyte {i.e., *ca.* −59 mV/log [(Li^+^)]; Figure 4B}. This feature was absent when CVs in 1 M TBAClO_4_ with no Li^+^ were measured. CVs measured with electrolytes containing Li^+^ beyond the range described above were quite distinct from others and hence were not considered (see Supplementary Figure S5 and the *Discussion* section for details). Nevertheless, within the reported concentration range, the Nernstian shift of the cathodic peak potentials indicates that this feature is associated with the LCET reaction. According to the Nernst equation, the *ca.* −59 mV/log [(Li^+^)] slope indicates a 1:1 stoichiometry of Li^+^ and e^−^; this stoichiometry is explicitly shown in Eqs 3, 4. We note these are essentially identical to Eqs 1, 2, but with the explicit notation of Li-to-electron stoichiometry and the redox-active metal oxide, WO_x_.

$${\text{WO}}_{x\;\left(s\right)}+n{\text{Li}}_{\;\left(\text{MeCN}\right)}^{+}+{ne}^{-}⇋{\text{Li}}_{n}W^{\left(6-n\right)+}O_{x\;\left(s\right)}$$ (3)

$$E=E{}^{\circ}-0.059\log\left(\frac{\theta_{{Li}_{n}W^{\left(6-n\right)+}O_{x}}}{\theta_{WO_{x}}}\right)-0.059\log\left(\left[{Li}^{+}\right]\right)$$ (4)

The peak potential of the cathodic Faradaic feature prior to that of the LCET reaction (labeled A in Figure 4A) did not scale with the Li-ion concentration (Supplementary Figure S6). As shown in Supplementary Figure S8, CVs of pristine MOF-808 also exhibited a single cathodic feature with a peak potential closely resembling that of feature A. Because Zr-nodes within the MOF are redox-innocent, we have ascribed this feature to be due to the reduction of the BTC linker.

The anodic Faradaic feature observed in all CVs of WO_x_@MOF-808 resembles the anodic Faradaic feature observed when bulk WO_3_ ∙ 2H_2_O was instead employed as an electrode. All CVs of WO_3_ ∙ 2H_2_O had a single wide feature with peak potentials *more similar than different* to that observed in CVs of WO_x_@MOF-808 measured in the same electrolyte (labeled C in Figure 4A); CVs of bulk WO_3_ ∙ 2H_2_O can be found in Supplementary Figure S9 in the SI.

Electrochemical treatments did not result in any changes to the PXRD pattern of the WO_x_@MOF-808 film, suggesting that the MOF backbone structure was retained. In contrast, the PXRD patterns of bulk WO_3_ ∙ 2H_2_O were distinct before and after an electrochemical treatment under Li-ion-containing electrolyte; see Supplementary Figure S3.

### 3.3 Analysis of LCET faradaic features

Here onwards, we will focus on the Faradaic feature with peak potentials scaling in a roughly Nernstian fashion with respect to the Li-ion concentration.

CVs of WO_x_@MOF-808 in the electrolyte containing 100 mM LiClO_4_ and 900 mM TBAClO_4_ were measured at scan rates between 10 and 100 mV/s (Supplementary Figure S7). The dependence of the peak currents vs. the measured scan rates is indicative of the LCET mechanism. When the logarithms of the two values linearly scale with a slope of 0.5, the LCET reaction rate is diffusion-controlled, where the “diffusion” of electrons (and in this case equimolar amounts of Li^+^) through the MOF lattice limits the overall reaction rate. When the slope is instead one, the reaction is kinetically controlled (Bard and Faulkner, 2001). As shown in Supplementary Figure S7, the logarithms of the peak cathodic current density (*j*_*p,c*_) values scaled against that of the log of the measured scan rate with a slope of ∼0.44, suggesting that the LCET reaction rate of WO_x_@MOF-808 is likely controlled by the diffusion of Li^+^ and e^−^ within the pore-confined WO_x_.

Compared to peak potentials, current densities had significantly larger sample-to-sample variation (see Supplementary Figure S4). These observations have been previously reported for MOF-based electrodes yielded from a simple drop-casting method (Chen et al., 2021). Error analysis due to these inconsistencies can be found in the Discussion section. Subtraction of background capacitive current led to somewhat more consistent *j*_*p,c*_, as described above in the scan rate dependence studies. The LCET Faradaic feature after the background subtraction was used to estimate the total amount of electroactive WO_x_ to be 11 ± 6 nmol/cm^2^. With the separate ^1^H NMR measurement determining the total amount of deposited WO_x_@MOF-808, *ca.* 0.2% of total WO_x_ on the electrode was found to be electroactive (see the SI for the details).

An ideal Faradaic feature exhibits a full-width-at-half-maximum (FWHM) value of 90 mV (Laviron, 1974; Laviron, 1979). At all concentrations measured in this work, the LCET Faradaic feature exhibited FWHM values of 120–150 mV (Supplementary Table S1). This contrasts with the wide FWHM values observed in all CVs of WO_3_ ∙ 2H_2_O with values well above 200 mV (see Supplementary Figure S9). Implications of these values in LCET thermochemistry are elaborated in the Discussion section.

## 4 Discussion

### 4.1 Brief elaboration on the synthesis of WO_x_@MOF-808

**FIGURE 5:**
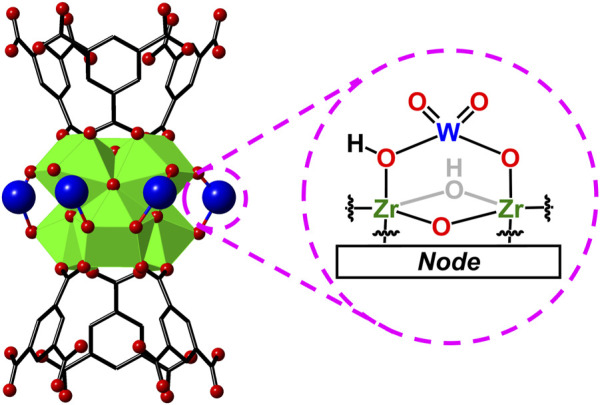
Proposed structure of tungstate on MOF-808 node, prior to acid condensation.

MOF-808 and many other Zr-based MOFs have high chemical stability over a wide range of pH (Bai et al., 2016; Howarth et al., 2016; Shi et al., 2023). Yet, our initial attempts in addition of more than the reported equivalence of Na_2_WO_4_ ∙ 2H_2_O to the MOF suspension resulted in a near-immediate decomposition of the MOF powder, indicated by the reaction mixture turning transparent. The presence of aqueous MOPS buffer at a pH of 7 was proven effective in bulk crystallinity retention. We ascribe the MOF decomposition due to the highly acidic tungstic acid (Pourbaix, 1974; Feng et al., 2017). With the procedure reported above, the successful incorporation of nearly all W within the reaction mixture into the MOF structure was achieved. We speculate that during the initial exposure of MOF-808 to the Na_2_WO_4_ solution, the [WO_4_]^2−^ binds to the Zr_6_ node, forming a W-oxo species like that proposed in Figure 5. We note the proposed structure of node-bound W-oxo species closely resembles those of crystallographically determined Mo^6+^-oxo species impregnated within MOF-808 and other Zr-based MOFs (Noh et al., 2016; Chen et al., 2022).

### 4.2 CVs of WO_x_@MOF-808

WO_x_@MOF-808 exhibited multiple Faradaic features; the peak potentials of each feature had a unique dependence with respect to the concentration of Li-ions within the electrolyte. In this report, we focused on the cathodic feature that scaled in roughly Nernstian fashion with an order of magnitude change in Li-ion concentration {i.e., ∼59 mV/log [(Li^+^)]}. This established the Li-to-electron stoichiometry involved in this reduction reaction to be 1:1 (Bard and Faulkner, 2001).

FWHM of a Faradaic feature is indicative of the chemical nature of the adsorbates formed and released during the heterogeneous redox reaction. An ideal FWHM value of 90 mV suggests that the adsorbates are chemically identical and do not laterally interact with each other (Gileadi, 1967; Laviron, 1974; Laviron, 1979). CVs of WO_x_@MOF-808 measured in 25–100 mM Li-ion concentrations revealed that the FWHM values of LCET Faradaic features were around 120 mV, which is quite similar to the ideal value. Scan rate dependence studies suggested that the observed LCET reaction is diffusion-controlled. Perhaps, the Li^+^/e^−^ pairs that are ‘adsorbed’ within the MOF pore-confined WO_x_ can diffuse to a site until the lateral interactions between the two sites are negligible. An increase in FWHM to 150 mV upon an increase in Li-ion concentration further supports this mechanism. Slight deviations from 90 mV may also arise from structural heterogeneity.

In general, CVs of tungsten and many other metal oxides that undergo LCET reactions exhibit multiple Faradaic features; some of them are reversible, while others are irreversible (Ding et al., 2014; Wu and Yao, 2017; Zhang et al., 2020; Tao et al., 2023; Wang et al., 2024; Zheng et al., 2024). This can be, at least in part, attributed to the bulk structural changes of metal oxides upon insertion of Li^+^ and other cations. For example, bulk WO_3_ in a monoclinic phase undergoes a dynamic structural transformation where the symmetry increases to orthorhombic, tetrahedral, and finally to cubic phase with increasing amounts of proton/lithium cations inserted within its lattice structure. Excess intercalation leads to an irreversible amorphization (He et al., 2016; Wang et al., 2016). We have also confirmed the irreversible structural change induced by Li-ion insertion by examining the PXRD pattern of WO_3_ ∙ 2H_2_O before and after an electrochemical treatment. Similar phase changes upon Li-intercalation were observed for titanium, manganese, nickel, and cobalt oxides and their complex mixtures (Qian et al., 2012; Lin et al., 2014; Kuppan et al., 2017; Myeong et al., 2018; Liu et al., 2019; Tao et al., 2023). The irreversible mass increase upon intercalation of Li^+^ or Na^+^ into TiO_2_ using electrochemical quartz crystal microbalance (EQCM) reported by Hupp and co-workers further supports this (Lyon and Hupp, 1995). While these structural changes have been documented solely for bulk metal oxides, it is conceivable that similar structural modulations occur with MOF-supported WO_x_, which leads to irreversibility.

### 4.3 LCET stoichiometry of WO_x_@MOF-808

The establishment of the stoichiometry of the reactants and products is fundamental to calculations of the electronic structure of different lithiated phases, and as demonstrated in the next section, thermochemical analysis (Mayer, 2023). LCET stoichiometry can be electrochemically established by measuring CVs in various concentrations of Li cations. This is a direct application of the approach in the PCET literature (Noh and Mayer, 2022). The CV-derived redox potentials at various proton activity yields the so-called Pourbaix diagram which can be used to determine the free energy of H-atom transfer (Pourbaix, 1974; Pourbaix, 1990). While the exact redox potentials of the MOF-confined WO_x_ cannot be determined due to its irreversibility, Figure 4B is a step toward a Pourbaix diagram for LCET reactions. For estimations of the free energy of LCET reactions and the associated errors, see Sections 4.4, 4.5.

CVs of metal oxides in various Li-ion concentrations required for the Pourbaix diagram are not reported often. This may be due to the complex phase transformation of many metal oxides as noted above. The lack of “well-defined” Faradaic features that can be ascribed to LCET further precludes this analysis; indeed, our attempts to measure LCET with bulk WO_3_ ∙ 2H_2_O synthesized analogously as to those MOF-confined also resulted in CVs with broad anodic features that did not exhibit any obvious trends with respect to Li-ion concentrations.

Nikitina et al. (2017) reported the well-defined, reversible LCET Faradaic features of LiMn_2_O_4_ to shift by 75 and 163 mV per unit change in log [(Li^+^)]. Using the Nernst equation (Eq. 2), this >59 mV shift suggests that more than one Li ions are involved per electron transfer. In the PCET literature, this is often referred to as the “super-Nernstian” dependence (Fleischmann et al., 2020). The additional positive charge has been speculated to be compensated by coupling the charge transfer reactions with anions within the electrolyte (Birss et al., 1991; Mayer, 2023). This super-Nernstian dependence is particularly prevalent for layered double hydroxides (LDHs) and other forms of layered metal oxides, which can intercalate ions (Burke and Lyons, 1986; Dincă et al., 2010; Lyons et al., 2012). The overall process, however, is quite complex involving partial or complete de-solvation of ions, intra-lattice diffusion, and others (Augustyn, 2017; Xu et al., 2022; Hu et al., 2023). In general, there lacks a fundamental theory that can correlate this super-Nernstian behavior to charge transfer thermochemistry. To the best of our knowledge, there are only a handful of reports explicitly determining the 1:1 cation-to-electron stoichiometry other than those in the PCET reactions; see the following references (Lyon and Hupp, 1995; Valdez et al., 2018; Saouma et al., 2019).

### 4.4 LCET thermochemistry of WO_x_@MOF-808 and comparisons with other redox-active metal oxides

Determination of thermochemistry, by definition, requires the chemical process to be at standard state and thermodynamically reversible. LCET reaction of WO_x_@MOF-808 reported here does not strictly follow these requirements.

Measurements beyond 250 mM in Li-ion concentrations proved unsuccessful due to the significant change in the Faradaic features. The apparent diffusion coefficient (D_app_) of Li-ions within the layered metal oxides significantly decreases at high concentrations, leading to a change in Faradaic features. These are typically observed at concentrations ≥1 M for bulk metal oxides (Nikitina et al., 2017; Finney et al., 2021). In the reported system, the Li-ions would have to de-solvate (at least partially) to diffuse into the MOF pores, even before reaching the redox-active sites (Sogawa et al., 2019). This may be the reason why 250 mM was the limit for WO_x_@MOF-808.

While the common reference electrode for Li-ion batteries and other LCET-related systems is Li metal, its potentials are unstable, varying as high as 0.5 V over the course of the reaction (Cengiz et al., 2021). Instead, we relied on the Fc^+/0^ redox couple as our reference in non-aqueous solvents (such as MeCN) due to their superior stability (Gagne et al., 1980; Gritzner and Kuta, 1984). Nevertheless, the standard potential of Li^+/0^ can be used to determine the free energy of lithiation, i.e., the addition of a “Li-atom.” In essence, this is treating the [WO^−^ ⋯ Li^+^] interaction much like a covalent bond and is analogous to the bond dissociation free energy (BDFE) readily reported for molecular/heterogenized species. This has proven powerful for direct comparison of thermochemistry between different substrates and reaction medium (Agarwal et al., 2021; Noh and Mayer, 2022). Because of the apparent irreversibility, free energy at standard state can only be estimated. We emphasize this by using the notation, ΔG_app,Li_ here onwards.

**FIGURE 6:**
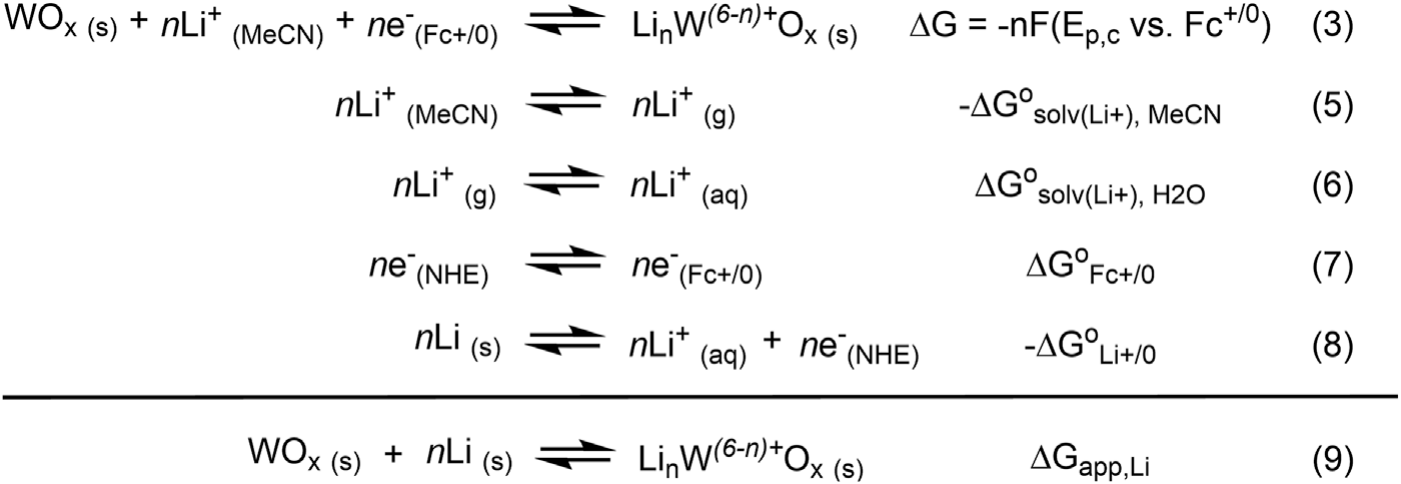
Scheme illustrating the derivation of ΔG_app,Li_.

All values shown in Figure 6 used for the estimation of ΔG_app,Li_ are at standard state [i.e., (Li^+^) = 1 M and 298 K in temperature]. The estimation of ΔG_app,Li_ requires the difference in solvation free energies of Li^+^ in H_2_O vs. MeCN (ΔG ° _solv(Li+), H2O_–ΔG ° _solv(Li+), MeCN_). Computationally derived solvation free energies of Li^+^ ions in H_2_O, MeCN, and many other protic and aprotic solvents are similar, typically ranging between −110 and −120 kcal mol^−1^. Though limited in reports, experimentally derived solvation free energies were within the same range (Carvalho and Pliego, 2015; Itkis et al., 2021). Thus, we estimated this difference in solvation free energies to be close to zero. The free energy of Fc^+/0^ and Li^+/0^ vs. normal hydrogen electrode (NHE) has been previously reported (Bard and Faulkner, 2001; Pegis et al., 2015). Using these values, for every electron transferred, ΔG_app,Li_ is estimated to be −36 ± 1 kcal mol^−1^.

To benchmark this value against other metal oxides that can undergo a net Li-atom transfer, we estimated the standard free energy of lithiation (ΔG ° _Li_) of tungsten, tin, titanium, manganese, nickel, and cobalt oxides available in the literature; here we assumed all redox processes involve 1:1 Li-to-electron stoichiometry as these reports lacked CVs in varying Li-ion concentrations. In general, the redox potentials of tungsten, tin, and titanium oxides that undergo LCET upon their *reductions* exhibit LCET Faradaic features within the range of 0.2–2 V vs. Li^+/0^, corresponding to ΔG ° _Li_ of −74 to −32 kcal mol^−1^ (Ding et al., 2014; Wu and Yao, 2017; Zheng et al., 2024). Manganese, nickel, and cobalt oxides that undergo *oxidative* LCET reaction have significantly higher ΔG ° _Li_ between −9 and +13 kcal mol^−1^ (Dahéron et al., 2008; Wang et al., 2013; Wang et al., 2024). ΔG_app,Li_ of WO_x_@MOF-808 is seemingly within the range, though the range is large. The ∼90 kcal mol^−1^ range in ΔG ° _Li_ highlights how LCET most likely has a complex dependence on the physical and chemical properties of metal oxides, including but not limited to the crystal structure, morphologies, and dopants.

**FIGURE 7:**
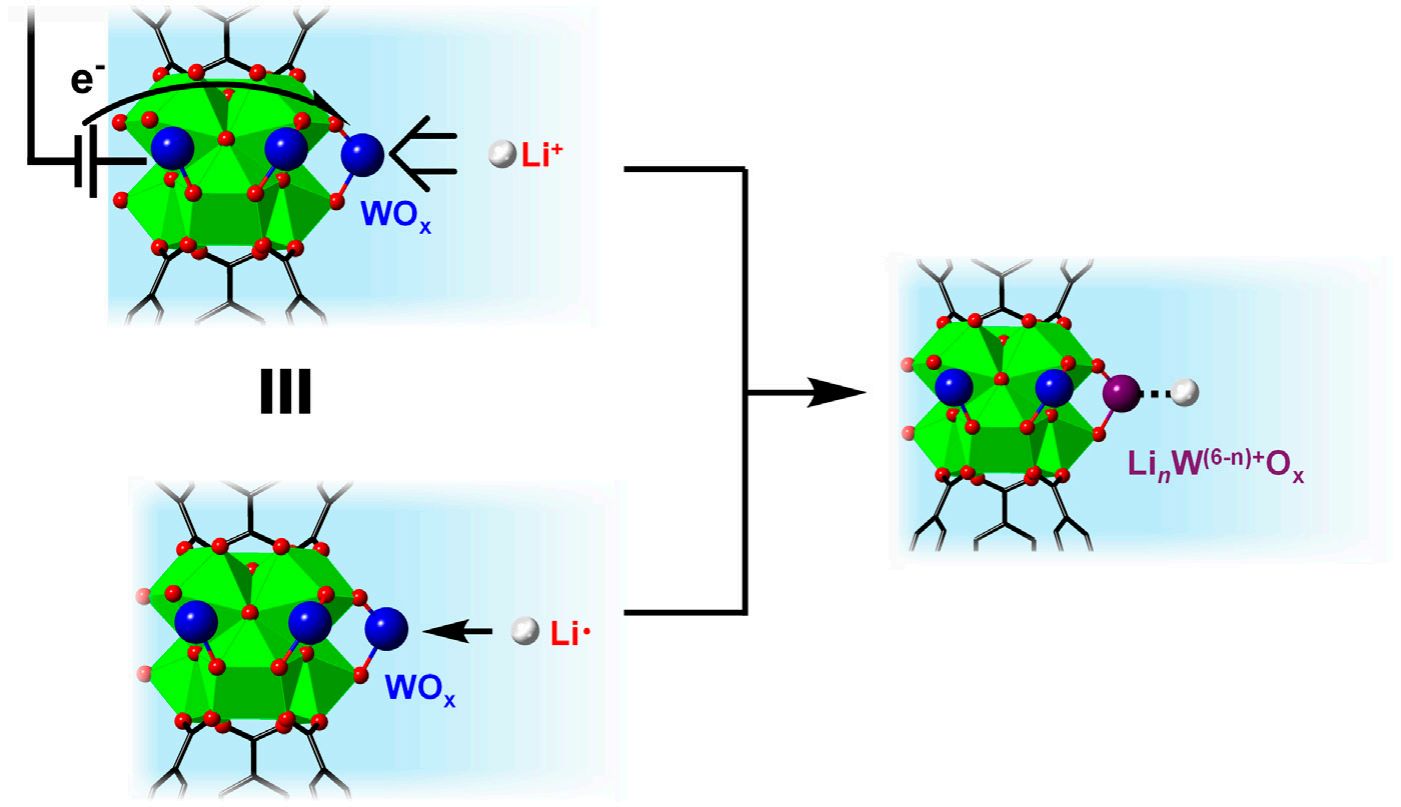
Schematic illustration showing the thermochemical analogy between LCET reaction vs. Li-atom addition on WO_x_ within MOF-808.

The above comparison highlights the power of using ΔG_app,Li_/ΔG ° _Li_ as one of the critical parameters to assess candidate electrodes for LIBs and others. These values are thermochemically equivalent to the reaction free energy of LCET (ΔG_LCET_; Figure 7). This is very much like the thermochemical equivalence between the free energy of hydrogenation vs. a PCET reaction with equimolar amounts of protons and electrons. Furthermore, ΔG_app,Li_/ΔG ° _Li_ is directly comparable between different substrates and reaction medium because this *should not* depend on the electrolyte compositions and their concentrations; hence ΔG_app,Li_/ΔG ° _Li_ is a much more robust parameter to standardize LCET thermochemistry.

### 4.5 Error analysis

We conclude this section by discussing the associated errors in estimating the free energy of LCET.

Thermodynamic reversibility is strictly required for the determination of ΔG ° (Agarwal et al., 2021). However, the ΔG_app,Li_ of WO_x_@MOF-808 was derived solely using the E_p,c_ values due to the observed irreversibility. Derivation of ΔG ° _Li_ requires the half-wave potential (E_½_) at various Li-ion concentrations. Here, we argue, however, that the derived ΔG_app,Li_ is still a reasonable approximation of the ΔG ° _Li_ of WO_x_@MOF-808. CVs of various metal oxides that exhibit *reversible* LCET Faradaic features have peak-to-peak separations (ΔE_p_) within the 100–200 mV range (Dahéron et al., 2008; Wang et al., 2013; Ding et al., 2014; Wu and Yao, 2017; Wang et al., 2024; Zheng et al., 2024). Thus, anodic and cathodic peak potentials are distinct from the E_½_ value by at most 100 mV, or 2.3 kcal mol^−1^. This error is significantly lower than many other errors inherently associated with ΔG ° _Li_. The solvation free energies of Li^+^ in MeCN vs. H_2_O, approximated to be similar in Section 4.4, can differ up to 10 kcal mol^−1^ (Carvalho and Pliego, 2015; Itkis et al., 2021). Even this difference is smaller than the wide range of ΔG ° _Li_ of various metal oxides (*vide supra*).

Between different samples, E_p,c_ values were consistent with standard errors <1 kcal mol^−1^. *j*_*p,c*_ values upon background subtraction were also somewhat consistent. However, the overall current had a large sample-to-sample variation. This large variation in currents has been previously observed for MOF-based electrodes yielded from a simple drop-casting method (Chen et al., 2021). While additions of conductive materials like carbon black and polymeric binders usually improve the consistency, these can convolute the thermochemical analysis. Carbon black is essentially the anode of LIBs and polymeric binders can slow the diffusion (Zhao et al., 2011). The observed inconsistency may also arise from the inhomogeneous distribution of hydrated WO_3_ within MOF-808 yielded after the acid condensation. Thus, for this report, we primarily focus on the thermodynamics of LCET and not its kinetics. Structural determination of the MOF-confined WO_3_ is beyond the scope of this work.

## 5 Conclusion and future outlook

Redox-active WO_x_ was successfully incorporated into the Zr-based MOF, MOF-808. In Li-containing electrolytes, CVs of the composite, WO_x_@MOF-808 exhibited multiple cathodic and anodic features, common for bulk metal oxides under similar electrochemical conditions. One of the reductive features scaled in a close-to-Nernstian fashion with respect to log [(Li^+^)], suggesting that this reductive process involves one Li cation per every electron transferred. Using this established stoichiometry, we estimated the free energy of lithiation to be roughly −36 kcal mol^−1^, which was comparable to the estimated values of other reported metal oxides, though the range was large (90 kcal mol^−1^).

Deposition of WO_x_ within MOF-808 resulted in an ancillary benefit of yielding a more “well-behaved” electrochemical system for thermochemical analysis. Faradaic features of bulk tungsten oxides and many other metal oxides do not exhibit Nernstian dependence with respect to Li-ion concentrations altogether precluding derivation of LCET thermochemistry. The reported success in estimating ΔG ° _Li_ encourages the exploration of other MOFs to examine the effects of the microenvironment within the MOF pores, which is our current focus. Some metal oxides exhibit reversible Faradaic features in a strictly oxygen-free environment (Lyon and Hupp, 1995). While this may be difficult to achieve using hydrated WO_x_ within hydrophilic Zr-based MOFs, exploration of other reaction conditions are also being currently examined. The deduced structure-thermochemistry relationships should become the cornerstone of next-generation battery design for a sustainable future.

We emphasize the robustness of the parameter, ΔG ° _Li_, in comparing candidate materials for Li-ion batteries. ΔG ° _Li_ is a solid-solid reaction of metal oxides and Li metal, and thus are independent of the solvents and electrolytes. This parameter should, therefore, be *intrinsically* related to the physical/chemical properties of the electrodes. This is also thermochemically equivalent to the minimal energy of LCET, and thus is an important parameter that must be considered for battery design.

## Data Availability

The original contributions presented in the study are included in the article/Supplementary Material, further inquiries can be directed to the corresponding author.
