# Supplementary material for: Lithium-coupled electron transfer reactions of nano-confined WOx within Zr-based metal–organic framework
Source: Front Chem. 2024 Jun 14;12:1427536. doi: 10.3389/fchem.2024.1427536 (PMC11214277; doi:10.3389/fchem.2024.1427536)
Supplement: Supplementary file 1 [file DataSheet1.pdf]

Supplementary Material

Lithium-Coupled Electron Transfer Reactions of Nano-Confined WOx within Zr-Based Metal–Organic Framework, MOF-808

Hafsa Abdul Ghuffar and Hyunho Noh*

*** Correspondence:** Hyunho Noh: hyunho.noh-1@ou.edu

1 General Considerations

1.1 Materials

All chemicals and materials in this work were used as received.

Fluorine-doped tin oxide glass (FTO; surface resistivity ~ 7 Ω/sq), Zirconyl chloride octahydrate (98%), 1,3,5-benzenetricarboxylic acid (H_3BTC; 95%), formic acid (≥88%), 3-(N-Morpholino) propanesulfonic acid (MOPS; ≥99.5%), sodium chloride (≥99.0%), acetonitrile (99.9%), Ferrocene (98%) and lithium perchlorate (≥95.0%) were purchased from Sigma-Aldrich. *N,N'*-Dimethylformamide (≥99.8%) was purchased from Supelco. Concentrated hydrochloric acid, Tetrabutyl ammonium perchlorate (98.0%), and sodium tungstate dihydrate (99+%) were received from Fisher Scientific. Ethanol (ACS grade) was received from Pharmco.

The two chemicals used for ^{1}H NMR, deuterium oxide (D_2O; 99.9 atom % D) and 40 wt% NaOD in D_2O (99+ atom % D) were both purchased from Sigma-Aldrich.

18.2 MΩ cm water (Millipore Synergy Water Purification System) was used to prepare an aqueous MOPS buffer. Concentrations of MOPS and NaCl were adjusted to 100 mM.

Zr-MOF-808 and bulk $WO_3 \cdot 2H_2O$ were synthesized according to the reported procedure.(Freedman, 1959, Liu et al., 2021)

1.2 Instrumentation

N_2-adsorption-desorption isotherms of Zr-MOF-808 and WO_x@MOF-808 were measured using the micropore analysis port of 3Flex (Micromeritics). Prior to the isotherm measurement, both samples were dried under a dynamic vacuum at 80 °C overnight and were further activated using VacPrep (Micromeritics) at 120 °C and at <50 mTorr. All Brunauer–Emmett–Teller (BET) areas were derived using the data set between P/P_0 = 0.005 – 0.1.(Howarth et al., 2017) For the isotherms and the density functional theory-calculated pore size distribution derived from the isotherms using the built-in 'N_2 – cylindrical Pores – Oxide Surface' model; see Figure 3 in the main manuscript.

All cyclic voltammograms (CVs) were measured using the CH instrument model 600D potentiostat. A WO_x@MOF-808-based electrode was prepared through a simple drop-casting method applied from previous reports (see Section 3 for details). Pt wire was used as a counter electrode. Ag/Ag^+ pseudoreference electrode was prepared according to the reported procedure.(Wise et al., 2020) At the end of all electrochemical measurements, a small amount of ferrocene was added to the electrolyte, and glassy carbon was used as a working electrode to measure its redox for the calibration of electrochemical potential.

Grazing incidence powder X-ray diffraction (PXRD) patterns were collected using Rigaku Smartlab equipped with a Cu Kα X-ray source. 2θ between 2 – 60° with a step size of 0.05°/min were used as the range with a fixed ω of 0.05°.

Scanning electron microscopy images and energy-dispersive X-ray spectra (SEM-EDS) were measured using the Zeiss Neon 40 EsB field emission instrument operated at 5 kV. Prior to the measurement, a small amount of Zr-MOF-808 or WO_x@MOF-808 was dispersed in acetone and drop-casted onto a polished Si wafer and was further coated with *ca.* 4 nm iridium using the EMS Quorum Q150 ES plus sputter coater.

^{1}H NMR spectra were collected using the Varian VNMR 400 MHz.

2 Physical Characterization of Zr-MOF-808 and WO_x@MOF-808

2.1 ^{1}H NMR Spectra

^{1}H NMR sample preparation follows that reported previously,(Ingram et al., 2024) using *ca.* 1 M NaOD to digest the MOF samples and DMSO as an internal standard. The ratios of the integrations measured three times using three freshly prepared electrodes were used to determine the average loading of WO_x@MOF-808.

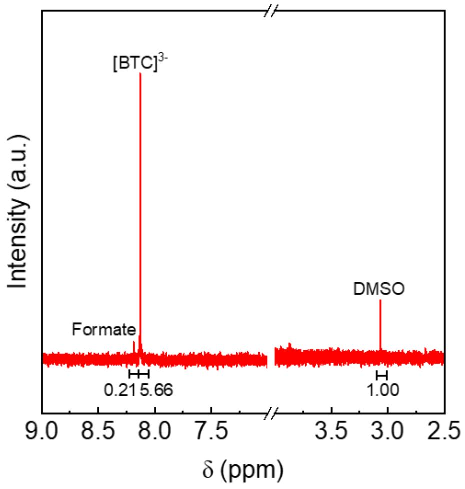

Supplementary Figure 1. Representative ^{1}H NMR spectra of digested WO_x@MOF-808.

Based on the three NMR spectra, we have determined the average loading of WO_X@MOF-808 to be 0.95 ± 0.13 $\mu mol_{WOX@MOF-808}/cm^2$ (or 5.725 ± 0.82 $\mu mol_{WOx}/cm^2$). Small amounts of formate were also observed, and this is a common observation for many Zr-based MOFs.

2.2 SEM Images and EDS Spectra

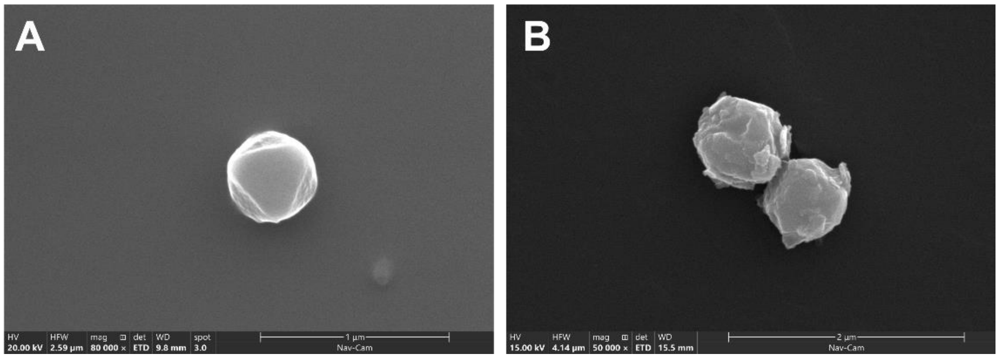

Supplementary Figure 2. SEM images of (**A**) pristine Zr-MOF-808 and (**B**) WO_x@MOF-808.

EDS collected in-situ to SEM measurements suggested that nearly all $[WO_4]^{2-}$ was incorporated into the MOF structure. As noted in the main manuscript, further addition of $[WO_4]^{2-}$ led to a MOF decomposition.

2.3 PXRD Patterns

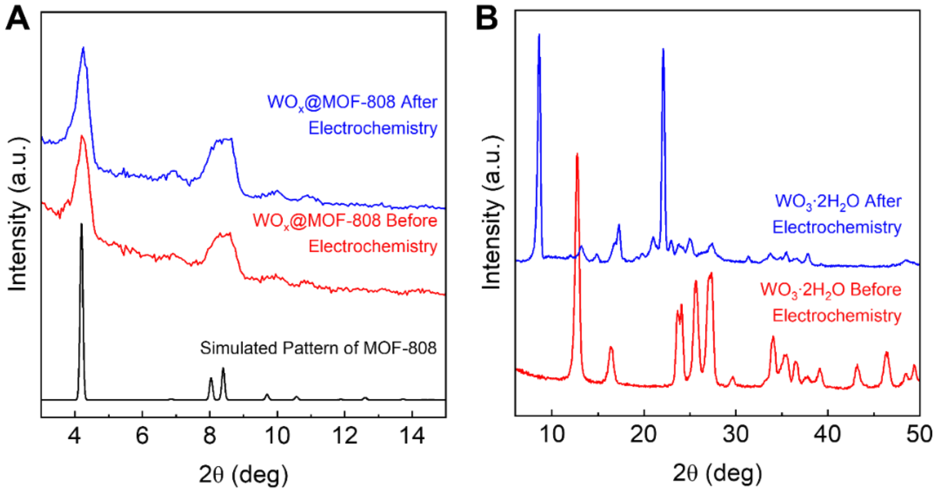

Supplementary Figure 3. PXRD patterns of (**A**) WO_x@MOF-808 or (**B**) $WO_3 \cdot 2H_2O$ on FTO before and after electrochemical treatment. For (**A**), the simulated pattern of pristine MOF-808 is also shown.

3 Details on Electrochemical Measurements

WO_x@MOF-808 was drop-casted onto FTO to yield the working electrode, using the modified procedure from that reported previously.(Ingram et al., 2024) Briefly, 12 mg of WO_x@MOF-808 was dispersed in 1 mL of acetone and was sonicated to yield a uniform suspension. Onto a 1 × 1 cm FTO, 10 μL of the suspension was drop-casted three times. Electrodes with bare Zr-MOF-808 or bulk $WO_3 \cdot 2H_2O$ was synthesized analogously, but with different masses to keep the number of moles of MOF or W^{6+} cation identical, respectively.

All errors presented in this section onwards are 1σ of duplicate measurements using freshly prepared electrodes.

3.1 Estimation of Electroactive Amount of WO_x within WO_x@MOF-808

The Faradaic feature associated with lithium-coupled electron transfer (LCET; feature highlighted as B in Figure 4A in the main manuscript) was integrated to determine the amount of electroactive WO_x. By considering the average of all CVs measured in this report, and using the following equation, we have determined the average amount of electroactive WO_x was determined to be 11 ± 6 nmol/cm^2; *i.e.*, based on the ^{1}H NMR results, this indicates that *ca.* 0.2% of all WO_x within the MOF network are electroactive. In eq. S1, Q, n, F, and N refer to charge, number of electrons, the Faraday Constant (96485 C/mol), and the number of moles of electroactive WO_x, respectively.

$$Q = nFN \qquad (S1)$$

3.2 CVs of WO_x@MOF-808

3.2.1 Sample-to-Sample Variation

Electrodes yielded through the above method proved successful in measuring the CVs of WO_x@MOF-808 and yielding the Pourbaix diagram of the LCET reaction (Figure 4B in the main manuscript). The large sample-to-sample variation, however, precluded any detailed kinetic analysis. As shown in the figure below, the CVs of two identically prepared electrodes in an identical electrolyte (100 mM $LiClO_4$ and 900 mM $TBAClO_4$ in MeCN) were quite distinct. This large sample-to-sample variation has been reported previously for MOF-based electrodes synthesized via simple drop-casting method.(Chen et al., 2021) We prefer not to use carbon black or polymeric binders, which are otherwise used commonly in the literature, as they are active towards LCET or can significantly hinder diffusion.

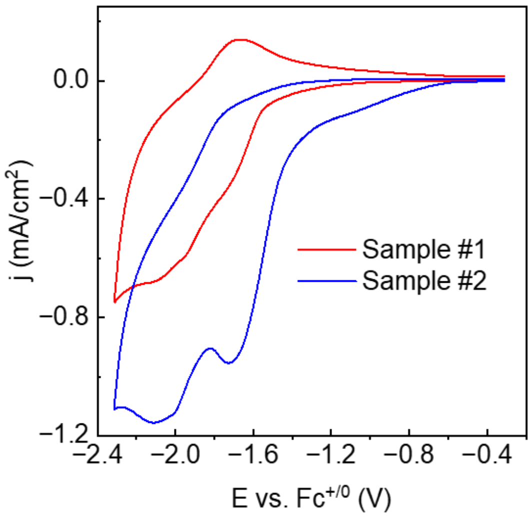

Supplementary Figure 4. CVs of two separate WO_x@MOF-808-based electrodes in 100 mM $LiClO_4$.

3.2.2 Beyond 25 – 250 mM Li^+ Ion Concentration

In this work, we focused on CVs measured in 25 – 250 mM Li^+-containing acetonitrile (MeCN)-based electrolytes. $TBAClO_4$ solution was used to keep the total ionic strength at 1 M. CVs in 1 M $TBAClO_4$ or 500 mM each of $LiClO_4$ and $TBAClO_4$ showed no Faradaic feature that can be ascribed to LCET; see below for the CVs. Thus, these concentration ranges were omitted from the analysis.

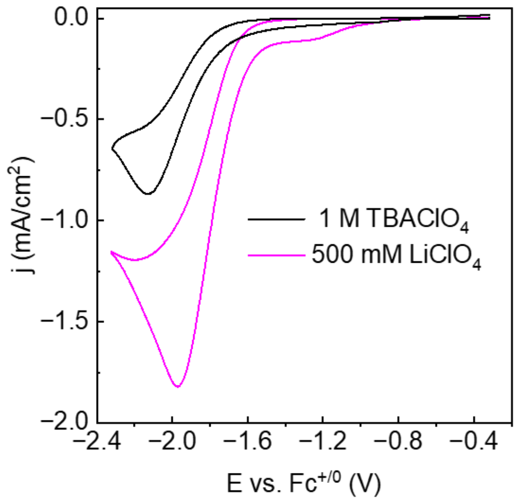

Supplementary Figure 5. CVs of WO_x@MOF-808 in 1 M $TBAClO_4$ or 500 mM $LiClO_4$.

3.2.3 Peak Potential vs. Li-ion Concentration of All Faradaic Features

The LCET Faradaic feature, labeled as B in Figure 4A in the main manuscript, scaled in a roughly Nernstian fashion with respect to Li-ion concentration. The other two Faradaic features (A and C in Figure 4A), however, exhibited either no dependence or a positive 'Pourbaix slope.' The latter cannot be explained by the simple Nernst equation and thus is beyond the scope of this work.

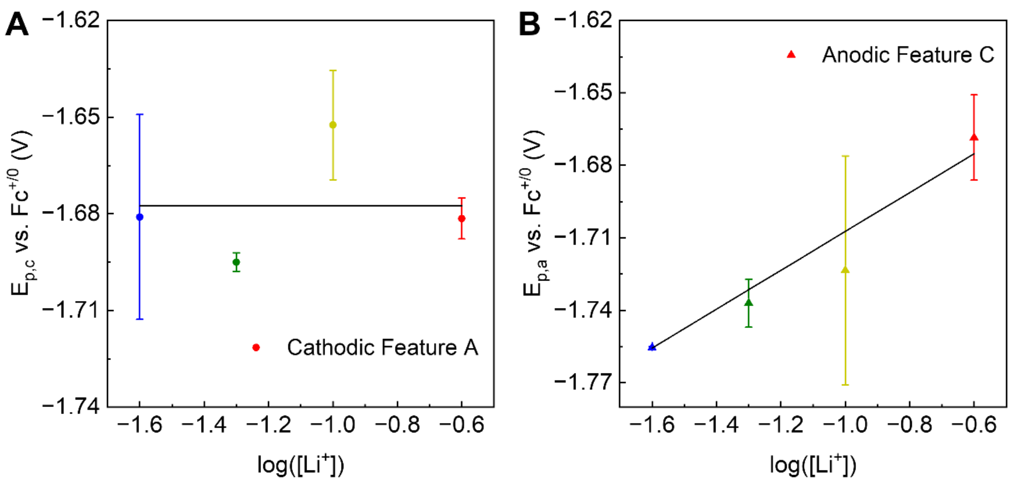

Supplementary Figure 6. Peak potentials vs. log([Li^+]) plots of (**A**) cathodic feature A and (**B**) anodic feature C. For details on feature labeling, see Figure 4A in the main manuscript.

3.2.4 Peak Potentials, Current Densities, and Full-Width-Half-Maximum of the LCET Faradaic Feature

Cathodic peak potentials ($E_{p,c}$), cathodic peak current densities ($j_{p,c}$), and full-width-half-maximum (FWHM) of the LCET Faradaic feature in various concentrations of Li^+ ion are reported in the table below. $j_{p,c}$ was derived after the subtraction of capacitive current; see the following reference for details. We note that these measurements were conducted at a scan rate (υ) of 100 mV/s. All errors are from two separate measurements using freshly prepared WO_x@MOF-808 electrodes.

Supplementary Table 1. $E_{p,c}$, $j_{p,c}$, and FWHM values of WO_X@MOF-808|FTO redox waves from CVs measured in the electrolyte containing various concentrations of Li^+-ion

$[Li^+]$ (mM)	$E_{p,c}$ vs. $Fc^{+/0}$ (V)	$j_{p,c}$ (μA/cm^2)	FWHM (mV)
25	–2.07(4)	32(6)	122(8)
50	–2.050(3)	43(2)	110(20)
100	–2.03(4)	30(10)	120(10)
250	–2.01(5)	100(30)	150(20)

3.2.5 Scan Rate Dependence

CVs of WO_x@MOF-808 measured in various scan rates (υ) at constant Li-ion concentrations should indicate the LCET mechanism. As shown below, the linear regression fit of the log(υ) vs. log($j_{p,c}$) resulted in a slope of 0.44 ± 0.07, indicating that the LCET reaction is diffusion-controlled. As noted in Section 3.2.1, the relatively large error bars on these figures are due to the sample-to-sample inconsistency.

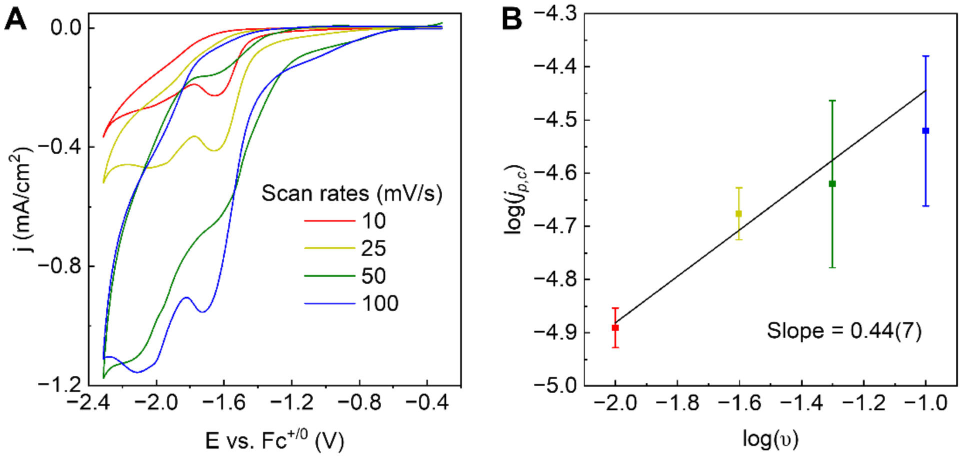

Supplementary Figure 7. (**A**) CVs of WO_x@MOF-808 in 100 mM $LiClO_4$ at various υ and (**B**) CV-derived log(υ) vs. log($j_{p,c}$) plot.

3.3 CVs of Zr-MOF-808 and $WO_3 \cdot 2H_2O$

CV using pristine Zr-MOF-808 as the working electrode was measured in 100 mM $LiClO_4$-containing electrolyte. As shown below, only one cathodic feature was observed, which was ascribed to the BTC linker reduction. We note that this reduction reaction was also observed for WO_x@MOF-808 (feature A in Figure 4A).

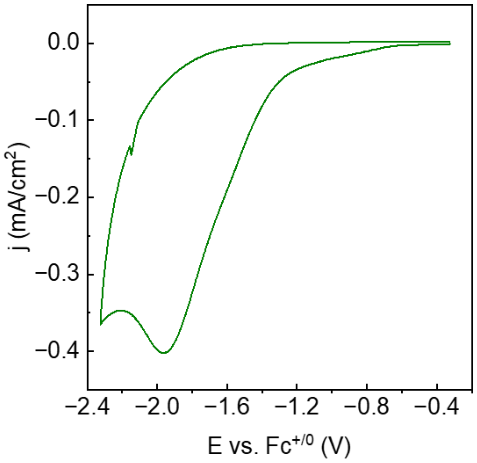

Supplementary Figure 8. CV of Zr-MOF-808 in 100 mM $LiClO_4$.

CVs of $WO_3 \cdot 2H_2O$ in various concentrations of Li-ion exhibited Faradaic features that did not exhibit any obvious trend with respect to Li-ion concentrations. Faradaic features were, in general, very wide with FWHM ~200-300 mV. Thus, further analysis of these CVs was not pursued in this work.

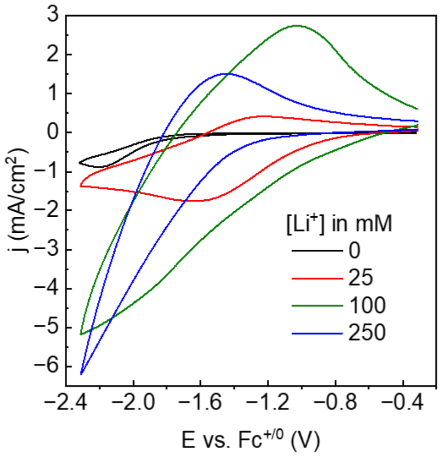

Supplementary Figure 9. CV of $WO_3{\cdot}2H_2O$ in 0 – 250 mM $LiClO_4$.

4 Details on Thermochemical Analysis

This section describes the details on how to convert the $E_{p,c}$ of LCET feature to the apparent free energy of lithiation, $\Delta G_{app,\,Li}$.

Many standard potentials required for thermochemical conversions are reported vs. normal hydrogen electrode (NHE). Thus, we first convert Li^+-ion solvated in MeCN to be that in H_2O using the difference in solvation free energies between two solvents, as shown below.

$$WO_{x\,(s)} + nLi^+_{(MeCN)} + ne^-_{(Fc+/0)} \rightleftharpoons Li_nW^{(6-n)+}O_{x\,(s)} \qquad \Delta G = -nF(E_{p,c}\ \text{vs. Fc}^{+/0}) \qquad (S2)$$

$$nLi^+_{(MeCN)} \rightleftharpoons nLi^+_{(g)} \qquad -\Delta G^o_{solv(Li+),\,MeCN} \qquad (S3)$$

$$nLi^+_{(g)} \rightleftharpoons nLi^+_{(aq)} \qquad \Delta G^o_{solv(Li+),\,H2O} \qquad (S4)$$

$$WO_{x\,(s)} + nLi^+_{(aq)} + ne^-_{(Fc+/0)} \rightleftharpoons Li_nW^{(6-n)+}O_{x\,(s)} \qquad \Delta G = 49\ \text{kcal mol}^{-1} \qquad (S5)$$

As noted in the main text, we estimated the solvation free energies to be similar between different solvents, given the previous reports.(Carvalho and Pliego, 2015, Itkis et al., 2021) Using $E_{p,c}$ extrapolated to standard state (*i.e.*, $[Li^+] = 1$ M) from Figure 4B, we calculated the ΔG of equation S5 to be 49 kcal mol^{-1}.

Next, the electrons involved must have the free energy references against NHE instead of $Fc^{+/0}$. The free energy of this reaction is already reported to be –15 kcal mol^{-1}.(Pegis et al., 2015)

$$WO_{x\,(s)} + nLi^+_{(aq)} + ne^-_{(Fc+/0)} \rightleftharpoons Li_nW^{(6-n)+}O_{x\,(s)} \qquad \Delta G = 49\ \text{kcal mol}^{-1} \qquad (S5)$$

$$ne^-_{(NHE)} \rightleftharpoons ne^-_{(Fc+/0)} \qquad \Delta G^o_{Fc+/0} = -15\ \text{kcal mol}^{-1} \qquad (S6)$$

$$WO_{x\,(s)} + nLi^+_{(aq)} + ne^-_{(NHE)} \rightleftharpoons Li_nW^{(6-n)+}O_{x\,(s)} \qquad \Delta G = 34\ \text{kcal mol}^{-1} \qquad (S7)$$

Finally, the redox potential of $Li^{+/0}$ can be used to derive $\Delta G_{app,\,Li}$.(Bard and Faulkner, 2001)

$$WO_{x\,(s)} + nLi^{+}_{(aq)} + ne^{-}_{(NHE)} \rightleftharpoons Li_nW^{(6-n)+}O_{x\,(s)} \quad \Delta G = 34\ \text{kcal mol}^{-1} \quad (S7)$$

$$nLi_{(s)} \rightleftharpoons nLi^{+}_{(aq)} + ne^{-}_{(NHE)} \quad \Delta G^{o}_{Li+/0} = -70\ \text{kcal mol}^{-1} \quad (S8)$$

$$WO_{x\,(s)} + nLi_{(s)} \rightleftharpoons Li_nW^{(6-n)+}O_{x\,(s)} \quad \Delta G_{app,Li} = -36\ \text{kcal mol}^{-1} \quad (S9)$$

5 References

Bard, A. J. & Faulkner, L. R. 2001. *Electrochemical Methods: Fundamentals and Applications,* New York, NY, John Wiley & Sons Inc.

Carvalho, N. F. & Pliego, J. R. 2015. Cluster-continuum quasichemical theory calculation of the lithium ion solvation in water, acetonitrile and dimethyl sulfoxide: an absolute single-ion solvation free energy scale. *Phys. Chem. Chem. Phys.,* 17**,** 26745-26755.

Chen, K., Downes, C. A., Schneider, E., Goodpaster, J. D. & Marinescu, S. C. 2021. Improving and Understanding the Hydrogen Evolving Activity of a Cobalt Dithiolene Metal–Organic Framework. *ACS Applied Materials & Interfaces,* 13**,** 16384-16395.

Freedman, M. L. 1959. The Tungstic Acids. *Journal of the American Chemical Society,* 81**,** 3834-3839.

Howarth, A. J., Peters, A. W., Vermeulen, N. A., Wang, T. C., Hupp, J. T. & Farha, O. K. 2017. Best Practices for the Synthesis, Activation, and Characterization of Metal–Organic Frameworks. *Chemistry of Materials,* 29**,** 26-39.

Ingram, Z. J., Lander, C. W., Oliver, M. C., Altınçekiç, N. G., Huang, L., Shao, Y. & Noh, H. 2024. Hydrogen-Atom Binding Energy of Structurally Well-defined Cerium Oxide Nodes at the Metal−Organic Framework-Liquid Interfaces. *Journal of Physical Chemistry C***, DOI:** 10.1021/acs.jpcc.4c02409

Itkis, D., Cavallo, L., Yashina, L. V. & Minenkov, Y. 2021. Ambiguities in solvation free energies from cluster-continuum quasichemical theory: lithium cation in protic and aprotic solvents. *Phys. Chem. Chem. Phys.,* 23**,** 16077-16088.

Liu, X., Kirlikovali, K. O., Chen, Z., Ma, K., Idrees, K. B., Cao, R., Zhang, X., Islamoglu, T., Liu, Y. & Farha, O. K. 2021. Small Molecules, Big Effects: Tuning Adsorption and Catalytic Properties of Metal–Organic Frameworks. *Chemistry of Materials,* 33**,** 1444-1454.

Pegis, M. L., Roberts, J. A. S., Wasylenko, D. J., Mader, E. A., Appel, A. M. & Mayer, J. M. 2015. Standard Reduction Potentials for Oxygen and Carbon Dioxide Couples in Acetonitrile and N,N-Dimethylformamide. *Inorganic Chemistry,* 54**,** 11883-11888.

Wise, C. F., Agarwal, R. G. & Mayer, J. M. 2020. Determining Proton-Coupled Standard Potentials and X–H Bond Dissociation Free Energies in Nonaqueous Solvents Using Open-Circuit Potential Measurements. *Journal of the American Chemical Society,* 142**,** 10681-10691.
